# MicroRNA Fingerprints Identify *miR-150* as a Plasma Prognostic Marker in Patients with Sepsis

**DOI:** 10.1371/journal.pone.0007405

**Published:** 2009-10-12

**Authors:** Catalin Vasilescu, Simona Rossi, Masayoshi Shimizu, Stefan Tudor, Angelo Veronese, Manuela Ferracin, Milena S. Nicoloso, Elisa Barbarotto, Monica Popa, Oana Stanciulea, Michael H. Fernandez, Dan Tulbure, Carlos E. Bueso-Ramos, Massimo Negrini, George A. Calin

**Affiliations:** 1 Department of Surgery, Fundeni Clinical Hospital, Bucharest, Romania; 2 Department of Experimental Therapeutics, The University of Texas M. D. Anderson Cancer Center, Houston, Texas, United States of America; 3 Department of Experimental and Diagnostic Medicine, Interdepartmental Center for Cancer Research, University of Ferrara, Ferrara, Italy; 4 Department of Hematopathology, The University of Texas M. D. Anderson Cancer Center, Houston, Texas, United States of America; 5 Department of Anesthesiology, Fundeni Clinical Hospital, Bucharest, Romania; Oregon Health & Science University, United States of America

## Abstract

**Background:**

The physiopathology of sepsis continues to be poorly understood, and despite recent advances in its management, sepsis is still a life-threatening condition with a poor outcome. If new diagnostic markers related to sepsis pathogenesis will be identified, new specific therapies might be developed and mortality reduced. Small regulatory non-coding RNAs, microRNAs (miRNAs), were recently linked to various diseases; the aim of our prospective study was to identify miRNAs that can differentiate patients with early-stage sepsis from healthy controls and to determine if miRNA levels correlate with the severity assessed by the Sequential Organ Failure Assessment (SOFA) score.

**Methodology/Principal Findings:**

By using genome-wide miRNA profiling by microarray in peripheral blood leukocytes, we found that *miR-150*, *miR-182*, *miR-342-5p*, and *miR-486* expression profiles differentiated sepsis patients from healthy controls. We also proved by quantitative reverse transcription-polymerase chain reaction that *miR-150* levels were significantly reduced in plasma samples of sepsis patients and correlated with the level of disease severity measured by the SOFA score, but were independent of the white blood counts (WBC). We found that plasma levels of tumor necrosis factor alpha, interleukin-10, and interleukin-18, all genes with sequence complementarity to *miR-150*, were negatively correlated with the plasma levels of this miRNA. Furthermore, we identified that the plasma levels ratio for miR-150/interleukin-18 can be used for assessing the severity of the sepsis.

**Conclusions/Significance:**

We propose that *miR-150* levels in both leukocytes and plasma correlate with the aggressiveness of sepsis and can be used as a marker of early sepsis. Furthermore, we envision *miR-150* restoration as a future therapeutic option in sepsis patients.

## Introduction

Sepsis represents a serious medical condition characterized by a whole-body inflammatory state called systemic inflammatory response syndrome, which is caused by a suspected or proven severe infection [1]–[3]. Severe sepsis occurs when sepsis leads to dysfunction of at least one organ or system due to hypoperfusion or hypotension, while septic shock is associated with refractory arterial hypotension despite aggressive fluid resuscitation and with multiple organ dysfunction syndrome. The criteria for diagnosis of sepsis and severity were established by the American College of Chest Physicians and the Society of Critical Care Medicine Consensus in 1992 and remain valid [2]. Despite recent advances in management, sepsis is still a life-threatening condition with a poor outcome and is the major cause of death among critically ill patients in intensive care units (ICUs) [3]. The physiopathology of sepsis continues to be poorly understood and, consequently, only a few specific therapies are available to treat this condition. Several scoring systems are used, including the sequential organ failure assessment (SOFA) score, but these systems evaluate clinical parameters related to the associated multiple organ dysfunction syndrome and do not include any factor involved in pathogenesis of the sepsis itself. Intraabdominal sepsis after abdominal surgery is responsible for approximately 13% of all ICU admissions [4], [5]. Studies have suggested that clearance of intraperitoneal sepsis may be beneficial when patients develop signs of intraabdominal sepsis after abdominal procedures [4]. However, the diagnosis of postoperative infections is difficult because clinical signs (e.g., pain, changes in level of consciousness, etc.) and laboratory findings, such as elevated acute-phase reactants (e.g., C-reactive protein), or fever are unspecific [5]. Therefore, new diagnostic markers could offer advantages over routine clinical and laboratory parameters in identifying postoperative patients with early-stage sepsis.

Recently, a new category of non-coding RNAs (RNAs that do not codify for proteins), named microRNAs (miRNAs), was found to be involved in the initiation, progression, and metastasis of any type of analyzed human cancer [6], [7]; miRNAs also play a role in other diseases, such as schizophrenia [8], diabetes [9], inflammatory bowel disease [10], and renal disease [11]. miRNAs are small RNAs that regulate expression of protein-coding genes by direct (sequence complementarity) interaction with and degradation of messenger RNAs (mRNAs) or by inhibition of protein translation [12]. miRNAs have been implicated in a wide array of cellular and developmental processes such as cell proliferation, apoptosis, and differentiation [12]. In a previous experimental study on mice, we demonstrated that *miR-155* and *miR-125b* play a role in innate immune response [13]. We found that lipopolysaccharide (LPS) stimulation of mouse Raw 264.7 macrophages resulted in the upregulation of *miR-155* and downregulation of *miR-125b* levels. The same changes also occurred when C57BL/6 mice were intraperitonealy injected with LPS. The data suggest that the LPS/tumor necrosis factor-alpha- (TNF-alpha) dependent regulation of *miR-155* and *miR-125b* may be implicated in the response to endotoxin shock, thus offering new targets for drug design [13]. As a continuation of our previous work, in the present study we interrogated miRNA expression in a set of sepsis patients by performing genome-wide profiling by microarray in leukocytes followed by quantitative reverse transcription-polymerase chain reaction (qRT-PCR) on plasma samples; we then compared the levels of miRNA expression in the sepsis patients with the miRNA levels in a series of healthy controls. Furthermore, we identified a plasma miRNA ratio (miR-150/IL-18) that can be used for assessing the severity of the sepsis.

## Materials and Methods

### Sepsis Patients and Healthy Controls

In the present study, we prospectively collected 24 samples from 17 sepsis patients (mean age +/− standard deviation (SD) = 55.4+/−17.13 y; 9 women, 8 men) and 32 samples from 32 healthy controls (mean age+/−SD = 49.2+/−20.12 y; 9 women, 23 men; *p* = 0.267 for age variation; *p* = 0.081 for gender) of peripheral blood leukocytes and/or plasma. All patients were admitted between November 2006 and March 2008 to the ICU at Fundeni University Hospital, Bucharest, Romania, after various types of abdominal surgeries (n = 8) or non-surgical related infections (n = 9) (**Table 1**). SOFA scores were assessed at day 1 and day 7; we also scored the outcome of each patient (alive or dead). The controls were healthy individuals selected from blood donors, patients' families, or research staff involved in the present study. “Healthy” was defined as the absence of any type of infection or known medical condition at the time of the study. All patients and controls were white Caucasians (according to medical records for patients and interview for controls).

**Table 1:** Clinical Data, SOFA Score, and Plasma Ratio miR-150/192 for Analyzed Sepsis Patients.

Patient ID	Sex	Age	Etiology	SOFA Day 1	Ratio miR-150/192 Day 1	WBC (x10^9^/L) Day 1	SOFA Day 7	Ratio miR-150/192 Day 7	WBC (x10^9^/L) Day 7	Type	Survival
S-1-76	F	76	Post-Surgery	1	0.442	15.7	NA	NA	10.2	SSP	alive
S-2-42	F	42	Post-Surgery	6	2.144	26.3	NA	NA	16.2	SSP	alive
S-3-78	F	78	Post-Surgery	1	2.307	25.4	NA	NA	14.4	SP	alive
S-4-78	M	78	Pulmonary infection	8	2.341	18.7	18	NA	26.4	SoP	alive
S-5-23	F	23	Pulmonary infection	18	3.704	26.6	NA	NA	NA	SoP	dead
S-6-62	M	62	Post-surgery	4	3.749	21.2	NA	NA	18.6	SSP	alive
S-7-55	M	55	Pulmonary infection	9	5.567	13.9	NA	NA	15.7	SSP	alive
S-8-39	M	39	Pulmonary infection	12	6.812	29.9	NA	NA	13.8	SoP	alive
S-9-62	M	62	Thymectomy Miastenia Gravis	6	8.176	8.3	5	15.363	9.2	SSP	alive
S-10-55	M	55	Post-surgery	13	8.203	3.2	NA	NA	NA	SSP	dead
S-11-73	F	73	Post-surgery	2	15.071	12.3	0	20.618	7.2	SP	alive
S-12-28	F	28	Acute hepatic failure	7	37.683	20.8	6	60.803	12.3	SSP	alive
S-13-43	M	43	Acute pancreatitis	2	45.776	19.9	0	0.528	16.6	SP	alive
S-14-43	F	43	Acute pancreatitis	1	47.310	15	0	23.970	NA	SP	dead
S-15-51	F	51	Intestinal occlusion	5	56.595	25.7	2	65.922	18	SSP	alive
S-16-65	M	65	Post-surgery	0	97.248	8.4	0	9.339	7.6	SP	alive
S-17-69^a^	F	69	Post-surgery	4	NA	14	6	48.940	10.2	SSP	dead

a Sample collected only at day 7.; SP, sepsis; SSP, severe sepsis; SoP, septic shock.

We obtained both peripheral blood leukocytes and plasma for 10 sepsis patients and 12 controls and only plasma samples for the remaining 7 sepsis patients and 20 controls. For seven of the patients, we collected plasma samples at two time points (days 1 and 7 after admission); for one patient, we collected a sample only at day 7 (**Table 1**). Peripheral blood leukocytes were obtained from 5 ml blood, and total RNA was purified using Trizol (Invitrogen, Carlsbad, CA). RNA quality was assessed by Agilent 2100 Bioanalyzer (Agilent Technologies, Palo Alto, CA), and only RNA with an RNA integrity number (a measure of RNA quality) higher than 7 was used for the expression-profiling study. Total RNA in plasma was isolated using Total RNA purification Kit (Norgen Biotek Corporation, Ontario, Canada) according to the manufacturer's instructions.

### Ethics Statement

For de-identification, the samples were codified S (for sepsis) and C (for controls) followed by a codified number to protect the privacy of individuals during all the further molecular study. All participants gave informed written consent to participate in this study and the samples were processed under approval of the Fundeni Hospital Ethics Committee.

### Genome-wide Human miRNA Expression Detection

Sixteen RNA samples from leukocyte (8 control, 8 sepsis) were hybridized on a human miRNA microarray (G4470A, Agilent Technologies). This microarray consisted of 60-mer DNA probes for 470 human miRNAs, sourced from the Sanger miRBase public database (Release 9.1). One-color miRNA expression was performed according to the manufacturer's procedure. Briefly, total RNA was obtained from samples by using the Trizol reagent (Invitrogen). Labeled miRNAs were obtained from 500 ng of total RNA through the ligation of a 5′-cytidine bisphosphate-Cy3 (pCp-Cy3, Agilent Technologies) group at the 3′-end of each miRNA. To enhance the T4 RNA-ligase (Promega, Madison, WI) efficiency, we had previously treated total RNA with alkaline phosphatase (Amersham, Piscataway, NJ) at 37°C for 30 min. Labeled miRNAs were purified on chromatography columns (Micro Biospin 6, Biorad Laboratories, Hercules, CA) and then hybridized on a microarray. Hybridizations were performed at 55°C for 17 h in a rotating oven. Images at 5-µm resolution were generated by a scanner (Agilent Technologies), and the Feature Extraction 9.5 software (Agilent Technologies) was used to obtain the microarray raw data.

### Microarray Data Analysis

Microarray results were independently analyzed in two distinct ways by MF in Ferrara, Italy and by SR in Houston, TX, respectively. First, using GeneSpring GX software version 7.3 (Agilent Technologies), we preprocessed data files with the plug-in for the Agilent Feature Extraction software results. Data transformation was applied to set all negative raw values at 5.0, followed by on-chip and on-gene median normalization. We filtered data for low gene expression so that only probes expressed (flagged as “present”) in at least one sample were kept; probes that did not change between samples, i.e., identified as having an expression value across all samples between median±1.5, were removed. Next, samples were grouped according to their status and then compared. Differentially expressed genes were selected as having a 2-fold expression difference between their geometrical mean in the two groups of interest (sepsis and control) and a statistically significant *p*-value (*p*<0.05) by analysis of variance statistics.

Independently, a second type of data analysis was performed by using extracted fluorescence intensity values (Agilent Feature Extraction) from all 16 hybridizations. The data were imported into Biometric Research Branch (BRB) array tool version 3.7.0 (http://linus.nci.nih.gov/BRB-ArrayTools.html) for subsequent microarray analysis. MiRNAs with less than 20% expression data with at least 1.5-fold (expression between median±1.5) change and probes with values missing from more than 50% of the arrays were removed, leaving 88 probes to be included in data analysis. Absent calls were assigned at a threshold of 7 (log_2_) before statistical analysis. Intensities were normalized using average factors scaled to the median array intensities over the entire array by using the median array as a reference. This filtering method was decided a priori to eliminate probes whose miRNA expression levels were thought to be unreliable. Class-comparison analysis using two-sided Student *t*-tests identified miRNAs that were differentially expressed between sepsis and control samples (*p*<0.05) and had a false discovery rate (FDR) of less than 9%. Cluster analysis was done with Cluster 3.0 (http://linus.nci.nih.gov/BRB-ArrayTools.html) and displayed using TreeView program (http://rana.lbl.gov/EisenSoftware.htm). Microarray raw data are MIAME compliant and were deposited in the public repository (ArrayExpress accession: E-TABM-713).

### qRT-PCR for miRNA Expression

MiRNA levels were detected by qRT-PCR using the TaqMan MicroRNA Assays (Applied Biosystems, Foster City, CA), according to the manufacturer's instructions. Experiments were performed in triplicate wells. To normalize the expression levels of target genes, we used U6B small nuclear RNA for the experiment performed with RNAs from leukocytes, while *miR-192* was used for plasma RNA normalization. The relative expression of each miRNA was calculated from the equation 2^-ΔCT^, where Δ C_T_ = mean Ct_miRNA_ – mean Ct_internal control_ (where Ct is the threshold cycle for a sample). Briefly, the relative abundance of each miRNA was calculated as the ratio of the value from sepsis to the value from controls, producing a fold-change value. Data for sepsis/control samples were compared using the two-sided Student *t*-test (*p*<0.05).

### MiRNA Target Prediction

We used two independent and complementary ways to predict miRNA targets. First, we used miRGen at http://www.diana.pcbi.upenn.edu/miRGen.html, which contains animal miRNA targets according to combinations of the widely used target-prediction programs miRanda, TargetScanS, and PicTar and experimentally supported targets from TarBase. Second, we used RNA22 at http://cbcsrv.watson.ibm.com/rna22.html, a pattern-based method for identifying miRNA-target sites and their corresponding RNA/RNA complexes, by using not only the 3′ untranslated region of the mRNA but also the full messenger sequence. We used the Database for Annotation, Visualization, and Integrated Discovery (http://david.abcc.ncifcrf.gov) to identify the pathway distribution of predicted targets. These pathways were presented according to the Kyoto Encyclopedia of Genes and Genomes (KEGG) database (http://www.genome.jp/kegg/). This is a database of biological systems, consisting of the genetic building blocks of genes and proteins; the identified pathways are composed by molecular interactions and reaction networks for metabolism, various cellular processes, and human diseases.

### Enzyme-Linked Immunosorbent Assay (ELISA)

Due to the availability of material, the levels of human tumor necrosis factor alpha (TNF-alpha, 12 control and 11 sepsis samples), human interleukin-10 (IL-10, 11 control and 20 sepsis samples), and human interleukin-18 (IL-18, 22 control and 18 sepsis samples) were measured by ELISA in undiluted plasma samples. Commercially available ELISA kits for TNF-alpha and IL-18 (R & D Systems, Minneapolis, MN) and IL-10 (BD Biosciences, San Jose, CA) were used according to the manufacturers' instructions. Results were read at an optical density of 450 nm using a Spectra Max Plus plate reader (Molecular Devices, Sunnyvale, CA). Measurements were performed in duplicate, and *p*-values were computed using the two-sided Student *t*-test (*p*<0.05).

### Statistical Analysis

The t-test on microarray data identified miRNAs that were differentially expressed between sepsis and control patients (*p*<0.01, FDR<0.09). We also identified miRNAs and cytokines that were differentially expressed in sepsis and control subjects for qRT-PCR data using t-test (*p*<0.05). Matlab 6.5 (www.mathworks.com) analysis was used for the Pearson's correlation. All statistical tests were two-sided, and statistical significance was defined as *p*<0.05.

## Results

### MiRNA Genome-Wide Profiling in Peripheral Blood Leukocytes Differentiate Sepsis Patients from Healthy Controls

**Figure 1:**
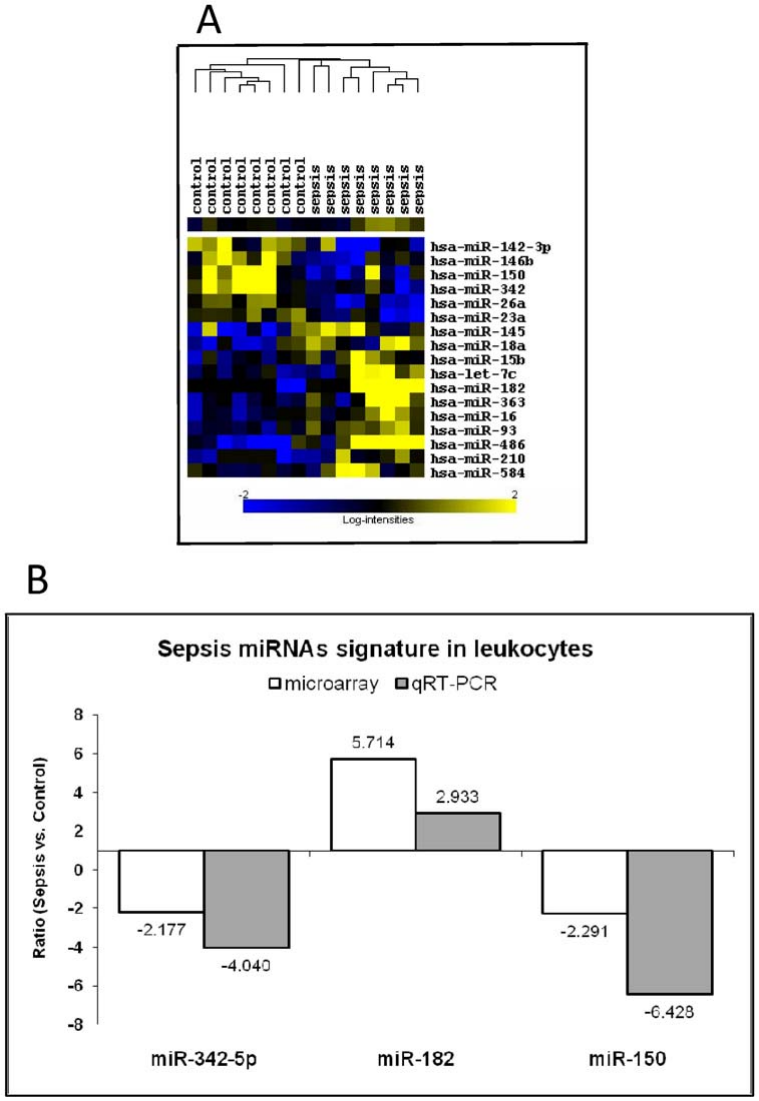
Leukocyte miRNA Signature Differentiates Early Sepsis Patients from Healthy Controls. (A) The cluster shows a perfect separation between the two classes of samples (sepsis *vs.* controls). The miRNA expression (log_2_) cluster shows differentially expressed genes as determined by *t*-test analysis. Yellow indicates high expression, and blue indicates low expression, relative to the median. (B) Fold change by qRT-PCR and array is shown for the leukocyte signature miRNAs. The most conspicuous differentially expressed miRNAs (sepsis *vs.* control) identified by both BRB array tools and GeneSpring GX software and also by qRT-PCR in leukocyte cells are shown.

To determine if miRNA patterns in patients with sepsis are significantly different from those in healthy controls, we first used microarray to compare the expression of 470 human miRNAs in leukocytes from eight patients at day 1 (first day in ICU) with expression profiles from eight healthy controls (**Figure 1**). As array data analyses could be performed by several methods that could reciprocally confirm each other, independent investigators performed this essential step using two different tools—the BRB array and GeneSpring GX software, respectively (see Materials and Methods). We found that a set of 17 miRNAs correctly differentiated 100% of the samples belonging to the two groups (sepsis and control), meaning that expression of these miRNAs was a good classifier for each category (**Figure 1A**). In both types of analyses, we identified four miRNAs— *miR-150*, *miR-182*, *miR-342-5p* and *miR-486* —whose expression levels differed by at least a factor of 2 between sepsis and healthy samples (*p*≤0.01; FDR<0.08): *miR-486* and *miR-182* were overexpressed, while *miR-150* and *miR-342-5p* were downregulated in sepsis patients. Of note, *miR-155* and *miR-125b*, the former upregulated, while the latter down-regulated by LPS stimulation of Raw 264.7 mouse macrophages [13], were not statistically significant dysregulated in the analyzed set of patients.

For independent confirmation, we performed a distinct type of quantification, qRT-PCR amplification for the active miRNA molecule in a set of 10 sepsis and 12 control leukocyte samples, including the 16 samples analyzed by the array. qRT-PCR was performed for *miR-150*, *miR-182*, *miR-342-5p*, and *miR-486* using the same total RNA as for the array; we used U6B as control. For all four genes we confirmed the same variation identified by the array, and in all instances, except for *miR-486*, with both methods the ratio was at least 2-fold between sepsis patients and controls (**Figure 1B**). Therefore, we were able to identify a miRNA signature that was associated with early stages of sepsis (on first day of admission to ICU). To get a higher confidence, we independently confirmed this signature using two types of statistical analysis and two types of miRNA quantification.

### *MiR-150* Levels in Plasma Are Significantly Reduced in Sepsis Patients Compared with Controls and Correlate with the Level of Sepsis Severity

**Figure 2:**
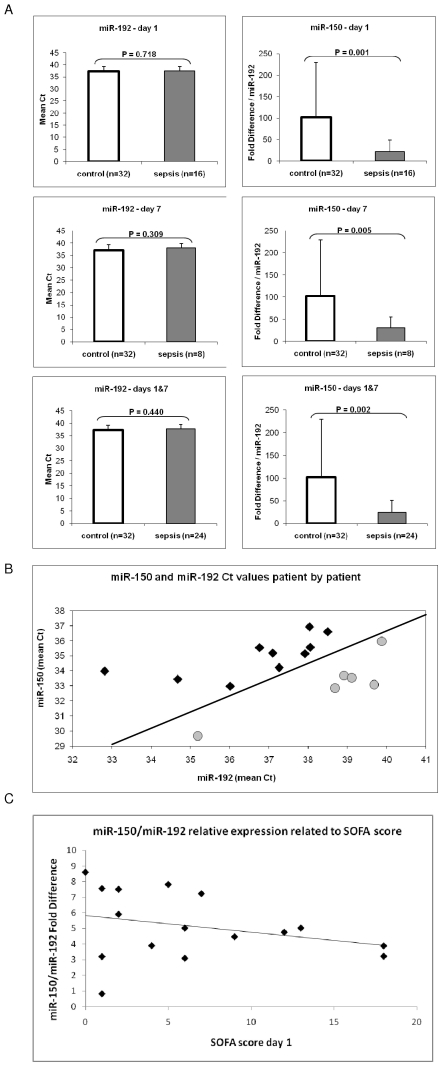
Plasma Level of *miR-150* is Significantly Lower in Sepsis Patients as in Controls. (A) Microarray analysis of *miR-192* levels in leukocytes found no variance in expression between patients and controls; qRT-PCR confirmed the same finding in plasma. Therefore, we used this miRNA as reference and normalized *miR-150* levels in respect to *miR-192* and found significant downregulation at both day 1 and day 7. Mean Ct plus standard deviation have been reported. The lower panel represents the combination of data from the upper two. (B) The *miR-150* and *miR-192* levels of 16 sepsis patients are plotted by their mean Ct values at day 1 after ICU admission. The round grey dots represent the patients with lower expression levels (2^-ΔCt^) and lower SOFA scores than the patients identified by square black dots. The grey shaded region indicates the threshold to classify *miR-150*, showing a clear division between samples with high and low expression levels based on *miR-192* as the normalizer. (C) The *miR-150* and *miR-192* levels of 16 sepsis patients are plotted by their miR-150/miR-192 relative expression values at day 1 after ICU admission related to the respective SOFA score. Interpolation line was reported.

Recently, miRNA expression in plasma was detected by using qRT-PCR techniques [14]. We focused for the present study on *miR-150*, as it has the highest levels of dysregulation in sepsis samples versus controls (**Figure 1B**). Therefore, we investigated the expression of *miR-150* in plasma by performing qRT-PCR on 24 sepsis (including 16 at day 1 and 8 at day 7) and 32 control samples. We initially used U6 and U6B for the selection of the normalizer gene, but since these RNAs were degraded in plasma we could not obtain reproducible results in triplicate experiments. Thereafter, we analyzed two miRNAs (*miR-192* and *let-7a*) that, according to the array data, had no variance in expression between sepsis samples and controls. The best reproducible results were obtained with *miR-192*; therefore, we used the expression of this miRNA as a reference value (**Figure 2A**). We found that *miR-150* was significantly down regulated in plasma from sepsis patients compared with controls at both days 1 and 7 at highly statistically significant values (*p* = 0.001 and *p* = 0.005, respectively) (**Figure 2A**). Therefore, plasma levels of *miR-150* (expressed as ratio with *miR-192*) reproduced the variations in leukocytes, and represent a reliable indicator of early sepsis. Importantly, we did not find any correlation between the plasma levels of *miR-150* and the number of leukocytes (expressed as white blood count, WBC) in sepsis patients (**Figure S1**), meaning that this downregulation is not just a biomarkers for the amount of circulating leukocytes. *MiR-182* and *miR-342-5p*, differentially expressed in leukocytes were studied also in plasma; the trend of variation was as in leukocytes for *miR-342-5p* and opposite for *miR-182*, and both of them were not significantly differentially expressed, probably due to the small number of sepsis samples with detectable expression (4 for *miR-182* and 3 for *miR-342-5p*).

In studying the expression of *miR-150* in plasma of the sepsis patients (qRT-PCR data), we noticed a correlation between the fold-difference value and SOFA score and/or associated severity of sepsis (grades labeled as sepsis, severe sepsis, and septic shock). Interestingly, by considering an 8.5-fold cutoff value for the expression ratio miR-150/miR-192 (meaning at least three amplification cycles' difference for the ΔCt between *miR-150* and *miR-192*) at day 1 (**Figure 2B**), we found that the 6 patients with higher ratios (15.07 to 97.24) had significantly lower SOFA scores than the 10 patients with lower ratios (0.44 to 8.20) (2.83+/−2.64 versus 7.80+/−5.41 mean ratio ± SD, respectively; *p* = 0.028) (**Table 1**). All three patients with septic shock had levels of *miR-150* lower than 8.5. Furthermore the miR-150/miR-192 relative expression was negatively correlated with the SOFA score (**Figure 2C**) and this ratio decreased from the sepsis to severe sepsis to sepsis shock patients (**Figure S2**). These data are in agreement with our initial observation that sepsis patients have lower levels of *miR-150* than healthy controls; in fact, the patient with the highest *miR-150/192* ratio (97.24) had the lowest SOFA score at both days 1 and 7. Further strengthening this conclusion, we did not find any significant differences in *miR-150* expression between day 1 and day 7 (43.98+/−29.35 versus 28.08+/−25.32; *p* not significant), as the SOFA score was quite similar for this limited set of patients (day 1+/−SD = 3.28+/−2.69 versus 1.86+/−2.61 for day 7; *p* not significant). Also, six of seven patients with measurements on both day 1 and day 7 had miR-150/miR-192 ratios concordant with their SOFA scores (**Table 1**). Therefore, consistently lower levels of *miR-150* were found in both leukocyte and plasma samples from sepsis patients than in healthy controls, and lower expression was associated with poor clinical condition as indicated by the SOFA score.

### *MiR-150* Expression Profile Is Correlated with Expression of Immune System Genes

**Figure 3:**
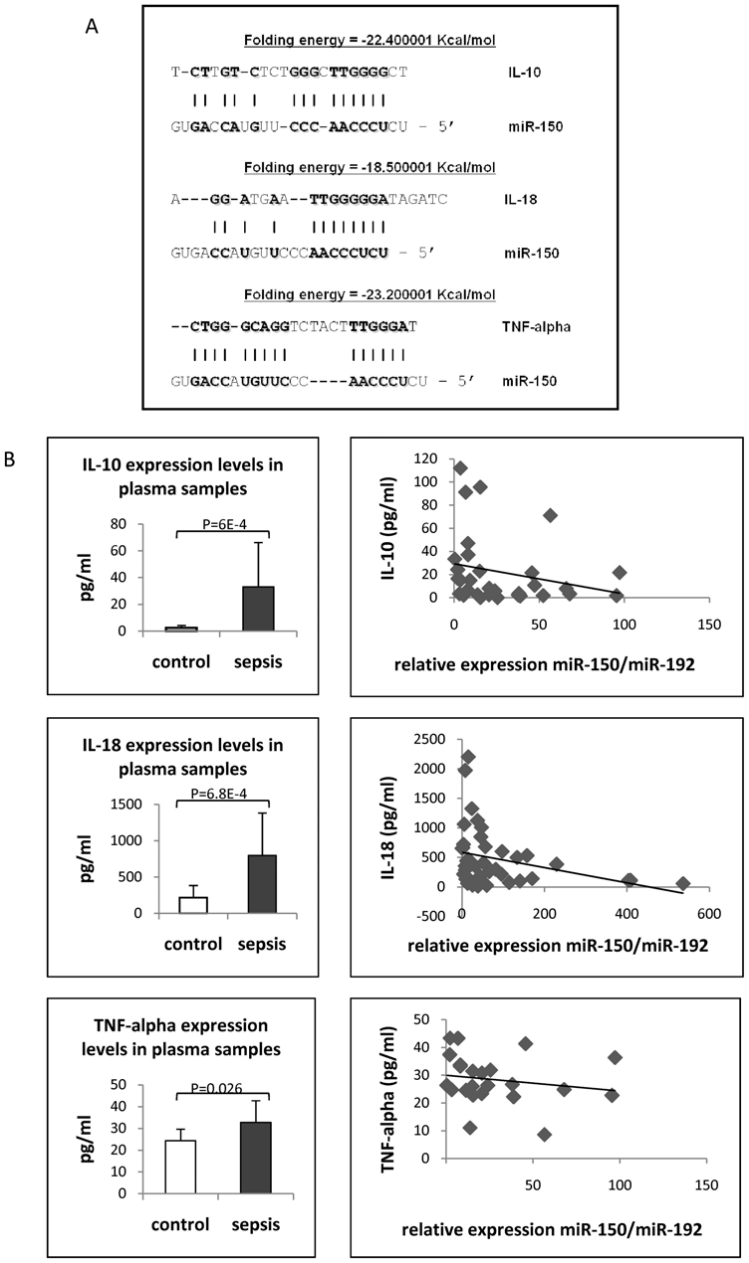
Negative Correlation Between Plasma Levels of *miR-150* and Cytokines. (A) The complementary sequence between *miR-150* and the mRNAs of TNF-alpha, IL-10, and IL-18 is shown. Complementary sequences are reported, as well as the relative folding energy between *miR-150* and mRNAs by using RNA22. (B) ELISA determination for plasma markers of sepsis and correlation with *miR-150* expression in plasma are depicted in the graphs. The left panels show IL-10, IL-18 and TNF-alpha measurements by ELISA in plasma, mean Ct +/− standard deviation have been reported. The right panels show the negative correlation between *miR-150* and IL-10, IL-18 and TNF-alpha, respectively. Values, on a patient by patient basis, have been reported for each cytokine studied.

As the next step, we tried to understand if the variations in *miR-150* expression were only bystanders for other unknown causal effects or if they were linked to the pathogenesis of this disease. One way to do this was to identify correlations in clinical samples between levels of *miR-150* expression and that of important protein-coding genes involved in the pathogenesis of sepsis. Dysregulation of miRNA levels would be anticipated to affect the translation of multiple protein-coding genes. Therefore, first we performed target prediction for *miR-150* by using miRGen and found that among the predicted targets, at least 20 genes were functionally related to immune system processes; among these was IL-18, which is reportedly increased in patients with sepsis [15]–[17]. Using the Database for Annotation, Visualization, and Integrated Discovery (http://david.abcc.ncifcrf.gov) to identify overrepresented pathways, we found that predicted *miR-150* targets were significantly (p<0.05) clustered in a few KEGG pathways, and the five most overrepresented pathways were all known to be involved in sepsis (**Table 2**): MAPK inhibition improves survival in endotoxin shock and prevents sepsis [18]; Wnt has a role in regulation of inflammation [19]; and insulin resistance [20], [21], ErbB (EGFR) [22], and mTOR [23] are related to immune and inflammatory response. As it is well known that IL-10 and TNF-alpha play an important role in immune response and are dysregulated in sepsis patients [24], [25], we checked by using an additional target prediction program (RNA22) to identify possible regions of interaction with *miR-150*. As shown in **Figure 3A**, *miR-150* has regions of complementarity to IL-10, TNF-alpha, and IL-18 mainly in the 5′-end, the most extended with IL-18. These data suggest possible direct interactions and, more important for our clinical study, suggest possible negative correlations between the expression of cytokines and *miR-150* in patient samples.

**Table 2:** The Most Overrepresented Pathways for *miR-150* targets According to KEGG *.

KEGG Pathway Term	Count	%	P value	GENE NAMES
hsa04010: MAPK signaling pathway	26	3.25%	4.73E-04	ACVR1B, ARRB2, CACNA1G, CACNB3, CACNG1, CACNG3, CACNG6, CACNA1D, DDIT3, DUSP16, DUSP3, ELK1, GADD45B, MAPK8IP1, MAPK9, MAP2K4, MAP3K12, MAP3K13, NFATC4, PAK1, PRKCA, PRKACG, SRF, TP53, AKT3, CRKL
hsa04150: mTOR signaling pathway	8	1.00%	0.009812	EIF4B, EIF4E, IGF1, RHEB, STK11, ULK2, AKT3, VEGFA
hsa04910: Insulin signaling pathway	14	1.75%	0.010769	CBL, CBLB, ELK1, EIF4E, FLOT2, MAPK9, PPARGC1A, PRKACG, PRKAR1A, RHEB, SLC2A4, SOCS1, AKT3, CRKL
hsa04012: ErbB signaling pathway	10	1.25%	0.018937	CBL, CBLB, ELK1, MAPK9, MAP2K4, PAK1, PRKCA, AKT3, CRKL, ERBB2
hsa04310: Wnt signaling pathway	14	1.75%	0.026812	APC, DVL2, EP300, FBXW11, FZD4, FZD7, MAPK9, NFATC4, PRKCA, PRKACG, PPP2CB, PPP2R1A, SOX17, TP53

* - Count, number of potential target genes in the pathway; %, percentage of pathway genes that are targeted by *miR-150*. The gene name is presented as in NCBI at http://www.ncbi.nlm.nih.gov.

**Figure 4:**
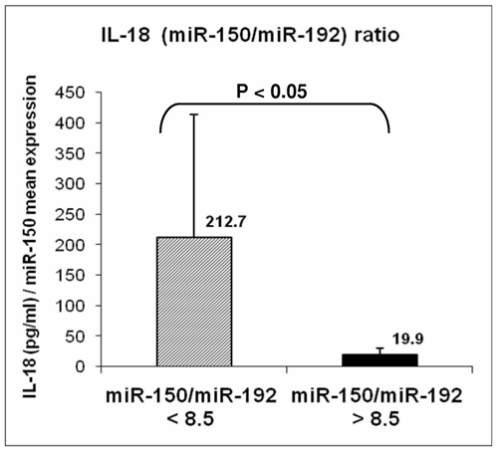
Ratio Between IL-18 and miR-150/miR-192 in Plasma of Sepsis Patients. A ratio between IL-18 ELISA assay expression and *miR-150* expression based on *miR-192* normalization was computed. Patients were separated into two groups related to the *miR-150* expression before the threshold was established (8.5). Patients with *miR-150* fold difference less than 8.5 provided an IL-18/miR-150 ratio statistically significantly higher than the group of patients with *miR-150* expression of more than 8.5 (P<0.05 by two-side *t*-test). Mean Ct +/− standard deviation have been reported.

Therefore, we performed ELISA to determine levels of pro-inflammatory TNF-alpha and anti-inflammatory IL-10 and IL-18 by using the same plasma samples in which the miRNA detection was performed. We found significant differences in expression levels between sepsis patients and controls (*p* = 0.026; *p* = 6.0E-04 and *p* = 6.8E-04, respectively) (**Table 3**), and these levels correlated with the plasma levels of *miR-150* in patients and controls (**Figures 3B**). These data confirmed the imbalance between pro-inflammatory and anti-inflammatory cytokines in our sepsis patients, further confirming the selection of the patients for this study. Supporting the separation of patients according to *miR-150* expression based on *miR-192* normalization levels in plasma, we found that IL-18 expression was markedly different between the two categories of patients (greater than 8.5 ratio versus less than 8.5 ratio). Therefore, we computed a ratio between IL-18 ELISA assay expression and *miR-150* expression: patients with miR-150/miR-192 fold difference less than 8.5 provided an IL-18/miR-150 ratio statistically significantly higher (more than 10 times higher, *p*<0.05) than the group of patients with miR-150/miR-192 expression of more than 8.5 (**Figure 4**). Although we did not ruled out a casual association by performing functional studies to prove the direct interaction between *miR-150* and one/several cytokines, these data suggest a possible functional correlation between *miR-150* plasma levels and cytokine expression in sepsis patients and that the plasma levels ratio for miR-150/interleukin-18 can be used for assessing the severity of the sepsis.

**Table 3:** Cytokine measurements from Septic Shock cases and Controls.

Cytokine	Control Subjects	Patients with Sepsis	P value
IL-10	6.55, 21 < DL	33.39 (5.79–112.1)	6E-04
IL-18	162.05 (15.23–532.60)	672.08 (107.55–2201.15)	6.8E-04
TNF-alpha	24.69 (11.05–31.85)	34.51 (26.01–44.16)	0.026

Definition of abbreviations: <DL = less than the detectable limit of 4 pg/ml for TNF-alpha or 5 pg/ml for IL-10 and IL-18.

Displayed are the plasma cytokine determinations quantified by chemiluminescence in picograms per millimeter (median+/−range). The actual number of analyzed patients and controls are as in Materials and Methods.

## Discussion

In the present prospective study we interrogated miRNA expression in a set of sepsis patients and found, by performing genome-wide profiling by microarray in leukocytes followed by quantitative reverse transcription-polymerase chain reaction (qRT-PCR) on plasma samples that *miR-150* is significantly down regulated in respect with healthy controls levels and can be used for assessing the severity of the sepsis.

This study is novel in several aspects. First, it addresses a significant medical condition, the sepsis, from a new perspective—the involvement of small non-coding RNAs. Sepsis is the major cause of death among critically ill patients in ICU. Total mortality for patients with acute postoperative sepsis is about 30%–35%; however, the mortality rate for patients with severe sepsis is over 50% [1], [26]. Consequently, severe sepsis is a disease in the need for critical care resources. We found that in sepsis patients the microRNoma (defined as the full spectrum of expressed miRNAs) is different from that in healthy controls, both in leukocytes (considered the cells involved in the immune disturbances characteristic of sepsis) and plasma on the first day of admission to the ICU. We found by microarray or qRT-PCR that the levels of three miRNAs — *miR-150*, *miR-182*, and *miR-342-5p* — are at least twice as dysregulated in leukocytes from patients than in those from healthy controls. Supporting these findings is a recent *in vitro* profile of the human leukocyte miRNA response to endotoxemia in which leukocyte RNA was isolated from venous blood samples obtained from three healthy male volunteers before and 4 h after LPS infusion and profiled for miRNA expression [27]. Five miRNAs consistently responded to LPS infusion, four of which were downregulated (*miR-146b*, *miR-150*, *miR-342*, and *let-7g*) and one of which was upregulated (*miR-143*) [27]. Also, *miR-150* was found to control c-Myb expression *in vivo* in a dose-dependent manner over a narrow range of miRNA and c-Myb concentrations, and this dramatically affected lymphocyte development and response [28]. These data further strengthen the functional significance of *miR-150* downregulation in sepsis patients. Furthermore, abnormal processing of *miR-21* transcript was recently reported in ICU patients suffering from sepsis-induced multiple organ failure [29], expanding the spectrum of miRNA alterations in sepsis.

Second, the recent identification of miRNAs in serum and plasma from healthy individuals and individuals with pathologic conditions, such as cancer, opens up the possibility of exploring miRNAs as biomarkers of disease [14]. To our knowledge, this is the first report of miRNA measurement in plasma from sepsis patients. We found not only that *miR-150* levels are significantly different in patients and healthy controls, but also that the levels of *miR-150* correlate with SOFA scores (but not with WBC). SOFA is a scoring system used to track a patient's status while in the ICU; it is based on six different scores, one each for the respiratory, cardiovascular, hepatic, coagulation, renal, and neurological systems [30]. We identified a ratio between the quantitative RT-PCR expression of *miR-150* and a nonvariable control *miR-192* that can be used to assess the severity of sepsis based on its correlation with the SOFA score. Additional candidates for large studies could be *miR-182* and *miR-342-5p* that we found differentially expressed in sepsis versus control leukocytes.

Finally, we revealed a new potential pathogenetic mechanism explaining some of the immune system dysfunctions in sepsis patients. The malfunction of regulatory mechanisms during sepsis can result in a loss of control of inflammation, eventually leading to profound immunosuppression and host damage [31]–[33]. Our study points to a miRNA regulation of pro- and anti-inflammatory genes involved in sepsis. We found that the expression levels of *miR-150* correlated with those of main immune response genes, such as TNF-alpha, IL-10, and IL-18. Furthermore, the putative spectrum of targets of *miR-150* is highly enriched in genes involved in immune system functions. Therefore, in addition to *miR-155* [34] and *miR-125* [35], *miR-150* could be one of the main regulatory miRNAs of immune function, and our study unraveled the clinical significance of the *miR-150* expression correlation with cytokine expression in patients with sepsis.

In conclusion, although the functions of most human miRNAs have yet to be discovered, miRNAs have emerged as key regulators of gene expression. The present data support the hypothesis that miRNAs are main regulators of the immune system, and abnormal expression has been found and can be used as a diagnostic and prognostic marker in immune disease. Sepsis is the newest addition to the long list of disease states proved by studies in patients to be linked to abnormal miRNA expression. One of the important regulators is *miR-150*, and this is significantly abnormally expressed in both leukocytes and plasma from sepsis patients. Our study is the first to identify a specific miRNA profile and to interrogate about the clinical significance of miRNA variations in sepsis patients. Due to the limited number of cases originating in the same center and to the bias toward intra-abdominal causes of sepsis, larger multi-institutional studies with higher numbers of patients will establish the final prognostic significance of our initial findings.

## Supporting Information

Figure S1 White blood count (WBC) and miR-150/miR-192 relative expression plot. No correlation was found in 23 sepsis samples (the WBC was missing for one sample) after standardization of *miR-150* relative expression values and WBC values meaning that miR-150/miR-192 ratio is not just a biomarker for presence or absence of circulating leukocytes in sepsis. The standard values were derived by subtracting the mean of the relative expressions for *miR-150* and *miR-192* and mean of the WBC, respectively from each individual relative expression value and WBC value, respectively, and then dividing the difference by the standard deviation, calculated for each one of the data series.
(2.61 MB TIF)

Figure S2 miR-150/miR-192 relative expression correlates with sepsis grade. The mean +/− standard deviation of miR-150/miR-192 fold difference related to sepsis grade (labeled as sepsis, SP, severe sepsis, SSP, and septic shock, SoP) is reported. As expected, *miR-150* relative expression is higher in low sepsis grade samples (SP). P values were not statistically significant, probably due to the limited number of analyzed samples.
(1.98 MB TIF)
