# Supplementary figures and images for: MicroRNA Fingerprints Identify miR-150 as a Plasma Prognostic Marker in Patients with Sepsis
Source: PLoS One. 2009 Oct 12;4(10):e7405. doi: 10.1371/journal.pone.0007405 (PMC2756627; doi:10.1371/journal.pone.0007405)

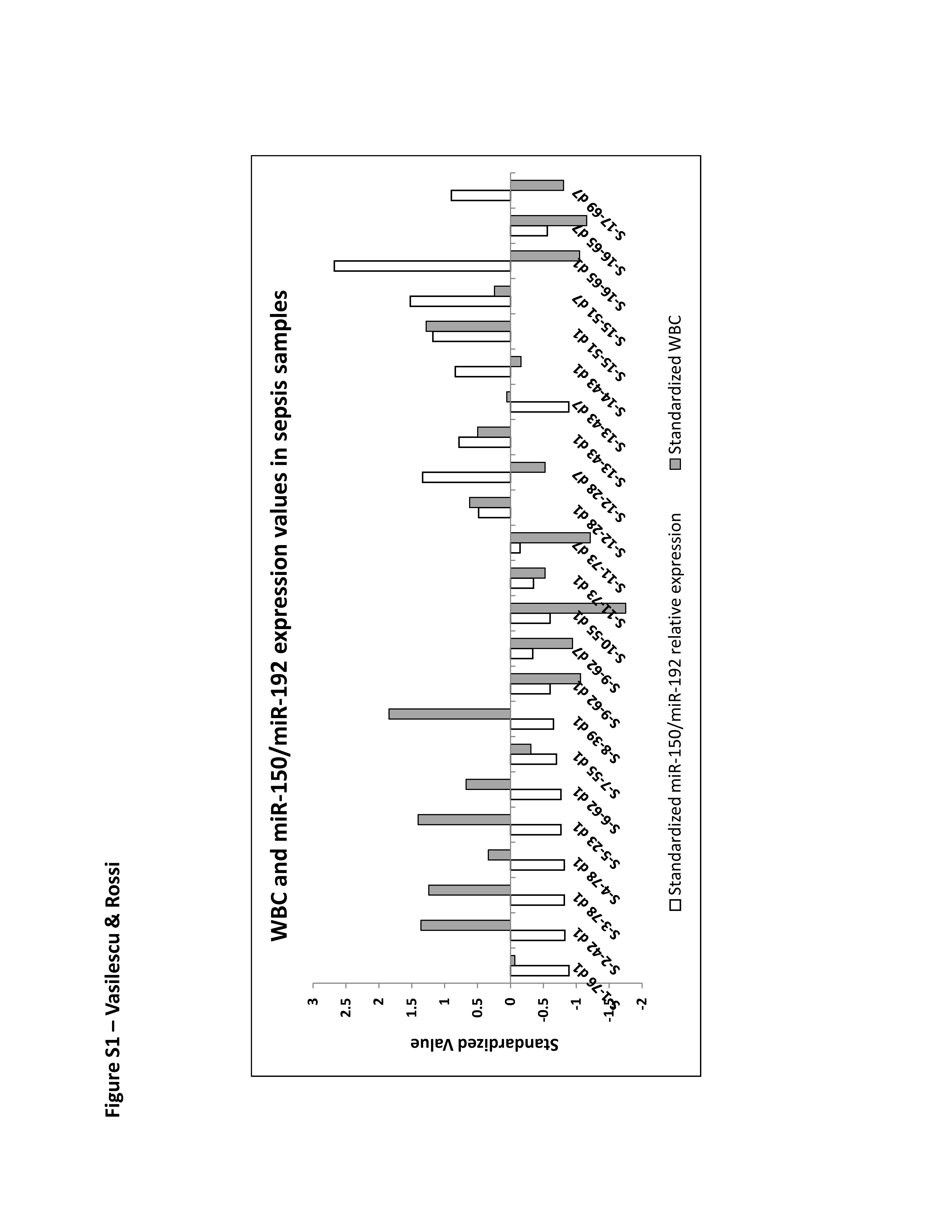

Supplement: Figure S1 — White blood count (WBC) and miR-150/miR-192 relative expression plot. No correlation was found in 23 sepsis samples (the WBC was missing for one sample) after standardization of miR-150 relative expression values and WBC values meaning that miR-150/miR-192 ratio is not just a biomarker for presence or absence of circulating leukocytes in sepsis. The standard values were derived by subtracting the mean of the relative expressions for miR-150 and miR-192 and mean of the WBC, respectively from each individual relative expression value and WBC value, respectively, and then dividing the difference by the standard deviation, calculated for each one of the data series. (2.61 MB TIF) [file pone.0007405.s001.tif]

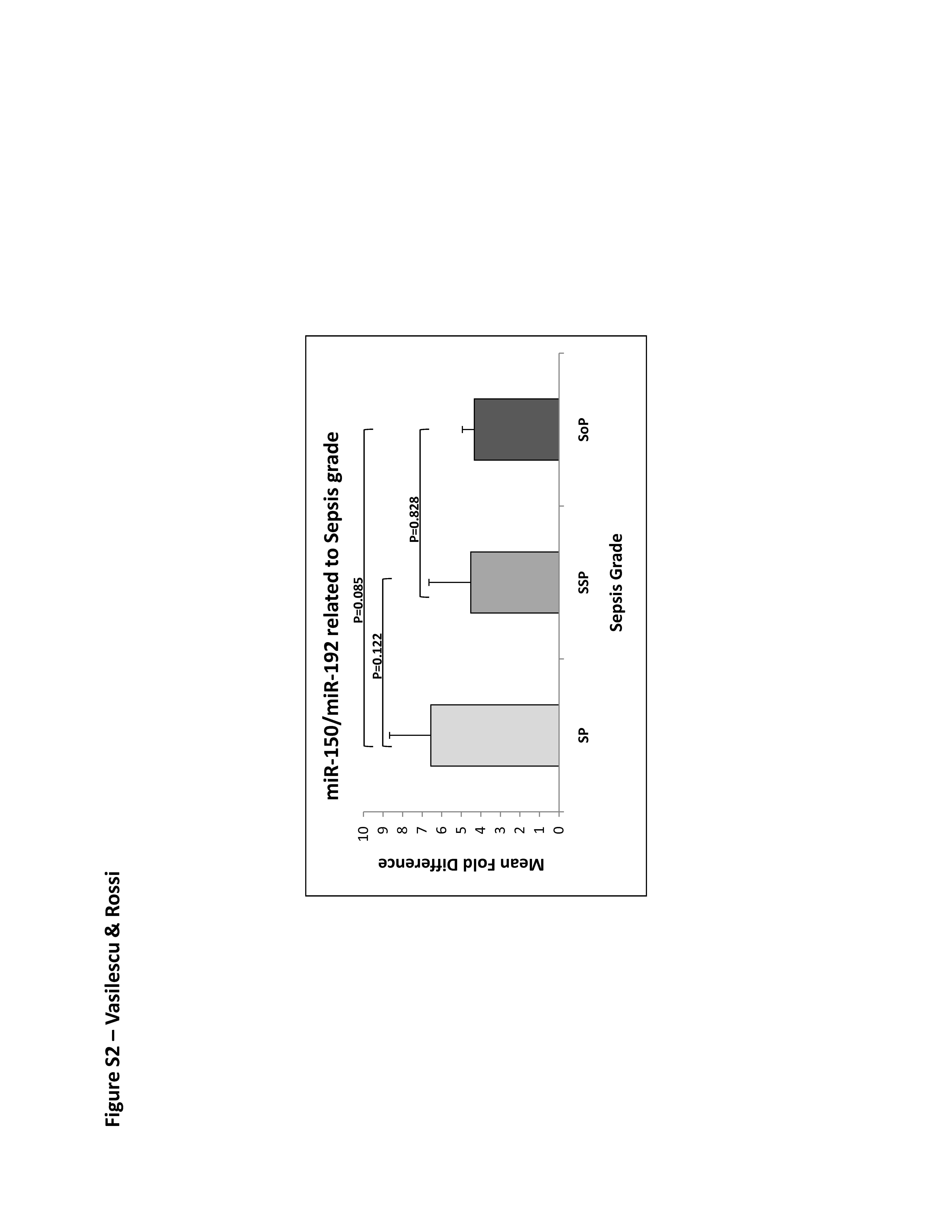

Supplement: Figure S2 — miR-150/miR-192 relative expression correlates with sepsis grade. The mean +/− standard deviation of miR-150/miR-192 fold difference related to sepsis grade (labeled as sepsis, SP, severe sepsis, SSP, and septic shock, SoP) is reported. As expected, miR-150 relative expression is higher in low sepsis grade samples (SP). P values were not statistically significant, probably due to the limited number of analyzed samples. (1.98 MB TIF) [file pone.0007405.s002.tif]
